# Untangling hairy cell leukaemia (HCL) variant and other HCL‐like disorders: Diagnosis and treatment

**DOI:** 10.1111/jcmm.18060

**Published:** 2023-12-14

**Authors:** Xavier Troussard, Elsa Maitre

**Affiliations:** ^1^ Hématologie CHU Caen Normandie Caen France

**Keywords:** hairy cell leukaemia variant, HCL‐V

## Abstract

The variant form of hairy cell leukaemia (HCL‐V) is a rare disease very different from hairy cell leukaemia (HCL), which is a very well‐defined entity. The 5th WHO edition (Leukemia, 36, 2022 and 1720) classification (WHO‐HAEM5) introduced splenic lymphomas/leukaemias including four different entities: (1) HCL, (2) splenic marginal zone lymphoma (SMZL) with circulating villous cells in the peripheral blood, (3) splenic lymphoma with prominent nucleolus (SLPN), which replaced HCL‐V and CD5 negative B‐prolymphocytic leukaemia (B‐PLL), and (4) splenic diffuse red pulp lymphoma (SDRPL). All these entities have to be distinguished because of a different clinical course and the need for a different treatment. The diagnosis can be challenging because of complex cases and overlap and/or grey zones between all the entities and needs integrating clinical, histologic, immunophenotypic, cytogenetic and molecular data. We review the diagnostic criteria including clinical, immunophenotypic and molecular characteristics of patients with HCL‐V and other HCL‐like disorders including HCL, SDRPL, SMZL, B‐PLL and the Japanese form of HCL. We also discuss the different criteria allowing us to separate these different entities and we will update the recent therapeutic options that have emerged, in particular the advances with chemoimmunotherapy and/or targeted therapies.

## 1 INTRODUCTION

The variant form of hairy cell leukaemia (HCL‐V) was recognized in 1980 by John Cawley et al.,^1^ who described two patients with a prominent splenomegaly and circulating villous cells with prominent nucleoli in the peripheral blood (PB). The splenic histology showed a predominant red pulp involvement and was similar to hairy cell leukaemia (HCL). There was no monocytopenia and the bone marrow could be easily aspirated without evidence of fibrosis. The authors proposed the term of HCL type II for this variant form, which was later designed as prolymphocytic variant of HCL. HCL‐V was subsequently included as a provisional entity in the group of splenic B‐cell lymphomas/leukaemias (unclassifiable) in the 4th edition of the World Health Organization (WHO) classification and its revised form. The 5th WHO edition^2^ classification (WHO‐HAEM5) introduced splenic lymphomas/leukaemias including four different entities: (1) hairy cell leukaemia (HCL), (2) splenic marginal zone lymphoma (SMZL) with circulating villous cells in the PB, (3) splenic lymphoma with prominent nucleolus (SLPN), which replaced HCL‐V and CD5 negative B‐prolymphocytic leukaemia (B‐PLL), and (4) splenic diffuse red pulp lymphoma (SDRPL). On the contrary, B‐PLL and HCL‐V were maintained in the clinical International Consensual Classification (ICC).^3^ De novo B‐PLL, recognized by Galton et al in 1974, is extremely rare: most of circulating abnormal lymphoid cells are medium‐sized prolymphocytes with a round nucleus, a prominent central nucleolus and a moderately condensed chromatin.^4^ The interest of regrouping HCL‐V and B‐PLL remains a subject of debate. *Because we focus on HCL‐V, we have chosen to use in this review the term HCL‐V rather than SBLPN*.

HCL is a well‐defined and recognized entity since 1958, with clonal mature late B cells expressing CD11c, CD25, CD103 and CD123. B‐raf proto‐oncogene (*BRAF* gene) (7q34) is composed of 18 exons: the mutation BRAFV600E occurs in exon 15 at position 1799, in which thymine and adenine are exchanged, leading to valine (V) being substituted by glutamic acid (E) at codon 600 (V600E) of the BRAF protein.^5^ Identified initially in all HCL cases, the mutation was subsequently detected in up to 95%–100% of HCL cases.^6^ By contrast, HCL‐V, SDRPL, SMZL, B‐PLL along with HCL‐Japanese variant (HCL‐JV), we will name after HCL‐like disorders, have less defined immunophenotypic and genetic characteristics. In all these entities, *BRAF* is wild type (WT). Mitogen‐activated protein kinase kinase 1. (MAP2KI) mutations can be identified in up to half of HCL‐V cases. There is a considerable overlap between all HCL‐like disorders and the possibility of progression/transformation from one entity to another makes difficult the accurate diagnosis in real life. Splenectomy could be required for a pathological diagnosis^7^: in SMZL, the splenic histology shows an expansion of the splenic white pulp with a biphasic, monophasic or atrophic pattern. Conversely, a primary and predominant involvement of the red pulp was identified in HCL, HCL‐V or SDRPL. In a cohort of 49 patients, who underwent splenectomy for SMZL (33 patients), HCL‐V (9 patients) or SDRPL (7 patients), either at diagnosis (30 patients) or after treatment (19 patients), the diagnosis after the histologic examination of the spleen changed in 26% of cases (5/19), from SMZL to SDRPL in 2 patients, chronic lymphocytic leukaemia (CLL) to SDRPL in 2 patients and from lymphoplasmacytic lymphoma (LPL) to SMZL in the last case. The authors also showed that splenectomy could be therapeutically useful in patients with SDRPL. However, the mortality after splenectomy is estimated around 7%: In some cases, we have to give up from operating on the patient. Identifying exactly the disease without spleen histology remains a clinical challenge with the need for appropriate treatment. HCL‐V is very close to SDRPL but has a more aggressive clinical presentation, with no response to purine nucleoside analogues (PNAs) and could benefit from a treatment with immunochemotherapy. In patients with relapsed/refractory disease, new therapeutic approaches have to be validated, in particular new oral targeted drugs.

We review the diagnostic criteria including clinical, immunophenotypic and molecular characteristics of patients with HCL‐V and other HCL‐like disorders including HCL, SDRPL, SMZL, B‐PLL and the Japanese form of HCL. We also discuss the different criteria allowing us to separate these different entities and we will update the recent therapeutic options that have emerged, in particular the advances with the targeted therapies.

## 2 HCL‐V SHOULD NO LONGER BE RELATED TO HCL‐SPECIFIC CHARACTERISTICS OF HCL‐V/SBLPN PATIENTS

### 2.1 Epidemiology and characteristics of HCL‐V/SBLPN patients

HCL‐V is a rare mature B‐cell chronic lymphoproliferative disorder (B‐CLPD), with a number of new HCL‐V cases estimated in 2016 at 810 in the United States, while new HCL cases are higher and estimated at 1.100.^8^ After direct age adjustment to the 2000 United States standard population, the incidence rate of HCL and HCL‐V varies according to ethnicity from 0.8 per 100.000 person‐years for white men, 0.5 for Hispanic males, 0.4 for black men and only 0.3 in Asian/Pacific men.^8^ In our register, we diagnosed 21.879 patients with malignant hematologic disorders between 1996 and 2020: HCL diagnosis was retained in 155 patients, representing 0.71% of all hematologic disorders. HCL‐V represented less than 5% of all HCL cases. HCL‐V affects predominantly elderly patients with a median age of 71 years (48–92) in 52 HCL‐V patients,^9^ higher than that observed in HCL (62 years: 33–97)^10^ The male predominance is less marked than in HCL: in the Swedish Lymphoma register, the male to female incidence ratio ranged from 1.15 (95% CI 1.09–1.22) in follicular lymphoma to 5.95 (95% CI 4.89–7.24) in HCL.^11^ The clinical course of HCL‐V is variable but usually more aggressive than that of HCL: the median survival was 9 years,^9^ a survival significantly shorter than that of HCL which is itself identical to that of the general population.^12^ Fifteen per cent survived beyond 17 years. The aetiology of HCL‐V is unknown with no evidence for an association between exposure to pesticides, impregnating agents or organic solvents and the development of HCL‐V. The most frequent initial manifestations were splenomegaly in 85% of cases. Hepatomegaly is present in less than one third of cases, while peripheral lymphadenopathy and involvement of other organs are rare.

### 2.2 Morphology and peripheral blood examination

**FIGURE 1:**
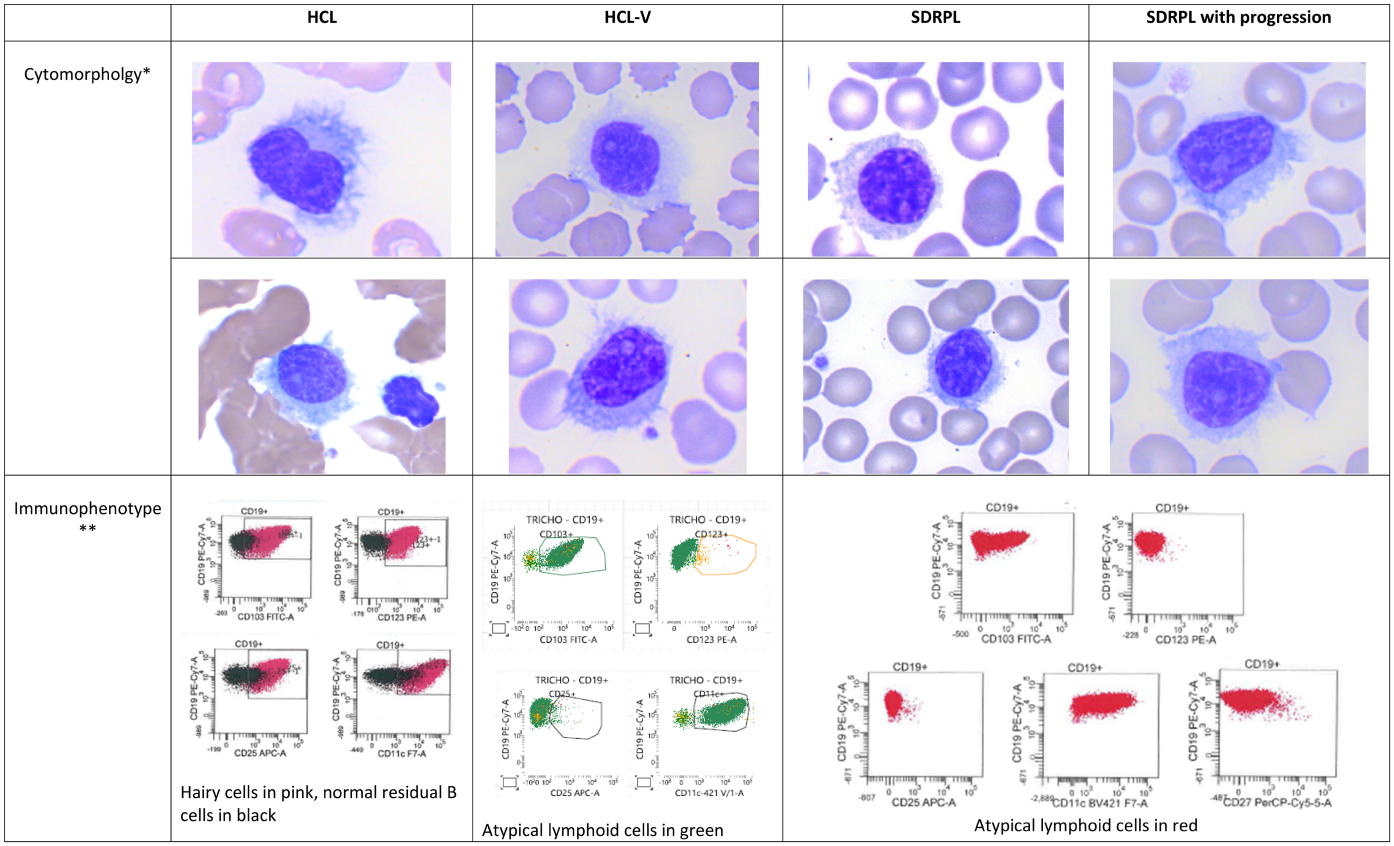
HCL: hairy cell leukaemia‐HCL‐V: variant form of HCL‐SDRPL: splenic diffuse red pulp lymphoma. *Cytomorphology of HCL: medium‐sized lymphoid cells, with round or kidney‐shaped nucleus, mature clumped chromatin, inconspicuous nucleolus, cytoplasm with long, fine and circumferential villi. May Grunewald Giemsa stain, ×1000. Cytomorphology of HCL‐V: medium‐sized lymphoid cells, with round or kidney‐shaped nucleus, mature clumped chromatin, prominent nucleolus, cytoplasm with well‐defined long, fine and circumferential or shaggy villi. May Grunewald Giemsa stain, ×1000. Cytomorphology of SDRPL: small‐sized lymphoid cells, with regular nucleus, mature clumped chromatin, inconspicuous nucleolus, cytoplasm with long and non‐polar villi. May Grunewald Giemsa stain, ×1000. Cytomorphology of SDRPL with transformation: medium‐sized lymphoid cells, with regular or kidney‐shape nucleus, mature but non‐clumped chromatin, prominent nucleolus, cytoplasm with long and non‐polar villi. May Grunewald Giemsa stain, ×1000. **Immunophenotype of HCL: CD103+, CD123+, CD25+ CD11c+. Immunophenotype of HCL‐V: CD11c + CD25‐ and CD123‐ (CD103+ in this case). Immunophenotype of HCL‐V: CD11c + CD27+ CD25− and CD123− (CD103 partially positive in this case).

This is an essential step, allowing us to analyse the number and the size of the abnormal lymphoid cells, the presence or the absence of a prominent nucleolus, the shape and the distribution of villi as well as the existence or not of lymphoplasmocytoid cells. Laboratory tests show a raised white blood cell (WBC) count. The absolute number and percentage of monocytes are normal. Anaemia and thrombocytopenia may be detected in one third of cases. Cytological features of HCL‐V and HCL are showed in Figure 1. The peripheral blood picture in HCL‐V is monomorphic, with a morphology of the abnormal lymphoid cells between hairy cells and prolymphocytes. The proportion of atypical lymphoid cells ranges from 20% to 95% and in most cases account for greater than 50% of mononuclear cells.^9^ The cells are medium to large in size and have an abundant basophilic cytoplasm with circumferential hair‐like cytoplasmic projections. The nucleus has a prominent vesicular nucleolus and a condensed chromatin. The majority of patients have interstitial bone marrow infiltration with hairy cells lying within sinusoids. In 10% of cases, the infiltration is mixed interstitial and nodular.

### 2.3 Immunophenotyping

The HCL‐V phenotypic profile is characterized by a clonal expansion of mature B cells arrested at a late stage of differentiation showing a strong light chain restricted surface immunoglobulin with a typical immunophenotype: bright expression of CD19, CD20 and CD22. An immunological score was proposed with one point given to each of the four markers (CD11c, CD25, CD103 and CD123) when it is expressed and no point when it is not expressed. A low score of 0 (26%), 1 (45%) or 2 (26%) was observed in HCL‐V, whereas the score was usually high: 3 or 4 in 98% of HCL cases.^13^ In HCL‐V, the expression of CD25 is negative in all cases at diagnosis and the expression of CD103 and CD123 is usually dim or negative. The expression of CD11c is positive and bright. In a series of 35 HCL‐V, aberrant antigen expression was reported, with a CD5 or CD10 positive expression in 3% of cases, CD23 in 14% and CD2 in 9% of cases. All HCL‐V cases were negative for annexin A1 (ANXA1) staining.^14^ In order to distinguish HCL‐V from HCL, the differential expression of CD43, CD81 and CD200 was used, with a median fluorescence intensity (MFI) lower for CD43, CD79b and CD200 but higher for CD81 in HCL‐V.^15^

HCL is characterized by bone marrow or extramedullary involvement by an abnormal population of light chain restricted B cells with a distinct immunophenotype comprised of CD11c, CD103, CD123 and CD25.^13^ HCL cells additionally co‐express CD19, CD20, CD22 and CD200 and are classically negative for CD5, CD10, CD23 and CD27 expressed in nearly 10%, 20%, 30% and 20% respectively.^16^ ANXA1 and phospho‐ERK expression are typically present in HCL but absent in HCL‐V. However, similar to other haematological malignancies, aberrant expression of atypical markers or loss of expression of typical markers may be seen. In our experience, an unusual phenotype was observed in 55/82 patients (HCL: 68 pts, HCL‐V/SDRPL: 5 pts and HCL‐like disorders unclassifiable: 9 pts) with a positive expression of CD5 in 7/68 HCL (10%) and CD23 in 22/68 (32%).^16^ It should also be noted CD25 is lost following treatment due to therapy‐related alterations and should not be relied upon for the diagnosis of HCL in patients who have already initiated therapy. The immunophenotypic profile of HCL may also provide insight into prognosis. The presence of CD38, was associated with substantially shorter time to next treatment (TTNT) with a difference in 3 years compared with those who were CD38‐. For the 27/43 patients who were CD38‐, TTNT was 83 months while it was only 42 months for the 16 patients who were CD38+. When analysing the 23 of the 43 patients that relapsed, TTNT of the 15 patients who were CD38‐ was 95 months and it was only 24 months for the 8 patients who were CD38 + .^17^

### 2.4 Unusual HCL‐V/SBLPN cases

When combining morphologic and immunophenotypic analysis, plasma cell leukaemia mimicking HVL‐V was observed in a few cases, with cells presenting a plasmacytoid appearance with basophilic cytoplasm and a positivity for CD138. CD103 and CD123 are usually positive and CD11c and CD25 can be negative.^18^ Composite lymphomas with CLL and HCL‐V were described. Although treatment of composite lymphomas may not be standardized, early detection of two distinct diseases and close follow‐up for disease progression may improve patient outcome.^19^

### 2.5 Cytogenetics and DNA copy number aberrations

Chromosomal abnormalities have been documented in limited cases.^9^, ^20^, ^21^ A complex karyotype was reported in 29% of cases.^22^ When using fluorescence in situ hybridization (FISH), a high frequency of deletion of 17p13/TP53 was found between 42% (5/12)^22^ and 100% (12/12)^21^ of HCL‐V. By high resolution genomic profiling, copy number alterations (CNAs) were identified in in 93% of HCL‐V (14/15) and in 86% of HCL (11/14), with a mean number of lesions per patient of 7.9 (0–27) in HCL‐V and only 3.4 (0–11) in HCL.^23^ The frequency of chromosome 5 abnormalities (trisomy, pericentric inversions and interstitial deletions) was identical in HCL‐V and HCL: 33% (5/15) in HCL‐V^23^ and ranged between 7%^23^ and 40%.^24^ Sixty‐six per cent of HCL‐V patients had 17p deletion with an unmutated (UM) *IGHV* profile compared with only 27% in patients with a mutated (M) profile.^23^

### 2.6 Molecular studies

The pattern of somatic hypermutations in the immunoglobulin heavy chain variable region genes (*IGHV*) appears heterogeneous in HCL‐V. The profile was UM between 22% (11/41)^25^ and 60% (6/10),^22^ while it was only 17% (5/30) in HCL. The UM profile, identified in 10% (6/58) of HCL cases^26^ was associated with a poor prognosis: bulky spleen (4 of 5: 80%), leucocytosis (3 of 5: 60%) and *TP53* defects (2 of 5: 40%). The patients also progressed rapidly after first‐line treatment, with a median event‐free survival (EFS) of 7.5 months.^26^

Compared with normal B cells, an over representation of *VH4‐34* was detected in HCL‐V and was used in 17% (7/41).^27^ *BRAF* was WT in all HCL‐V cases as well as *IGHV4‐34* positive (+) HCL. In a study including 53 HCL and 16 HCL‐V cases, 5/53 (9.5%) HCL and 8/16 HCL‐V (50%) expressed *IGHV4‐*34. *BRAF* was mutated in 42 (79%) HCL and was wild type (WT) in 11 (21%) HCL and all HCL‐V (16/16). All 13 *IGHV4‐34*(+) HCL were BRAF WT.^28^ In one other study, IGHV4‐*34* was used with a high frequency of 63.6% in primary vitroretinal lymphomas^29^ and significantly unevenly distributed, with 8/20 HCL‐V (40%) and 6/62 HCL (10%). Ninety‐three per cent of the *VH4‐34* rearrangements were observed in patients with an UM IGHV profile. Clinical features of VH4‐34+ patients were similar to those with HCL‐V and included higher white blood cell counts at diagnosis, lower response and progression‐free survival (PFS) after first‐line CDA and shorter overall survival (OS).^30^, ^31^

The identification of mitogen‐activated protein kinase kinase 1(*MAP2K1)* mutations is an argument for HCL‐V diagnosis but the mutation is detected in only 50% of cases. Activating mutations in *MAP2K1* gene (15q22.1‐q22.3) were found in *VH4‐34+* HCL (5/7 pts) and in HCL‐V (CD103+, CD25‐) either IGHV4‐34‐negative (−) (6/15 pts) or IGHV4‐34 − + HCL‐V (4/9 pts). In contrast, *MAP2K1* mutations were identified in only 1/20 cases of IGHV4‐34‐ ‐HCL patients.^32^ High prevalence of cyclin D3 (*CCND3)* and U2 Small Nuclear RNA Auxiliary Factor 1 (*U2AF1)* mutations were also identified in HCL‐V. In contrast to HCL, *CCND3* mutations were observed in 13% of HCL‐V cases, a frequency identical to that observed in SMZL and less than 25% in SDRPL. *CCND3* mutations involve the regulating PEST domain leading to cyclin D3 overexpression. Recurrent hotspot mutations of *U2AF1* encoding a protein belonging to the spliceosome were detected in 15% of HCL‐V.^33^ Tumour Protein p53 (*TP53)* mutations were identified in 30% of cases, and the survival at 5 years was just 11% in patients with *TP53* mutation versus 73% in those without^27^

Sequencing at diagnosis and relapse demonstrated the emergence of new mutations at relapse. In addition to the overexpression of cyclin D1, recurrent inactivation of the cell cycle inhibitor cyclin dependent kinase inhibitor 1B (*CDKN1B*/*p27)* was identified in more than 10% of cases.^34^ Inactivating Krüppel‐like Factor 2 (*KLF2)* mutations were observed in 15% of HCL.^35^ KLF2 is a transcription factor controlling the differentiation of multiple B‐cell subpopulations, including marginal zone B cells. Mutations of the genes of the epigenetic regulation genes were frequently observed, with mutations in the histone methyltransferase Lysine Methyltransferase 2 C (*KMT2C –* MLL3) occurring in 15% of patients and more rarely mutations in histone demethylase lysine demethylase 6A (*KDM6A*) or histone acetyltransferase CREB‐binding protein (*CREBBP*‐CBP). In other mutations in the chromatin remodelling complex family AT‐Rich Interaction Domain 1A (A*RID1A)*, *ARID1B* were also described. The myeloid differentiation primary response 88 *MYD88* ^L265P^ most commonly seen in lymphoplasmacytic lymphoma (LPL) was also detected in 23% of HCL (3/13).^36^

A recent study carried out at diagnosis in 10 HCL patients showed that in one out of two cases, mutations were identified in the non‐coding regions and in the promoter regions of the BCL6 transcription repressor *(BCL6)* and ZFP36 ring finger protein like 1 (*ZFP36L1)* genes Both genes generate somatic mutations (SHM) outside the target immunoglobulin genes. This study also showed a relative chromosomal stability and a phenomenon of kataegis (localized hypermutations) in particular in the 7q34 region involving the B locus of the T cell receptor^37^

### 2.7 Survival, transformation/progression

The course of the disease is usually chronic, with a prognosis worse than that of HCL, SMZL or SDRPL.^20^ In a series of 35 patients, three adverse factors emerged for a poor outcome: anaemia (<10 g/dL), older age (>70 years) and the presence of *TP53* mutations. One or more of these risk factors defined a high‐risk group (33% survival at 5 years) versus 100% in the low risk without any of these factors. The OS at 5 years was only 11% in patients with *TP53* mutations compared with 73% in those without it.^20^ Mutations of the *MAP2K1* gene were also associated with a poor prognosis for time to next treatment (11.5 months vs. 49.8 months) and PFS (10 months vs. 46.9 months).^35^

Four HCL‐V patients were reported with an initial diagnosis of HCL‐V that progressed to diffuse large B‐cell lymphoma (DLBCL). In one patient, HCL‐V diagnosis was confirmed by splenectomy. The patient also presented *U2AF1* mutations, while BRAF was WT. DLBCL diagnosis was later confirmed by biopsy of a lymph node. The use of *IGHV4‐34* was identified in the spleen and in the lymph node, confirming in that case a clonally related high‐grade DLBCL.^38^

## 3 HCL‐V/SBLPN IS VERY CLOSE TO SPLENIC DIFFUSE RED PULP LYMPHOMA AND SPLENIC MARGINAL ZONE LYMPHOMA

The characteristics of patients with HCL‐V and other HCL‐like disorders are listed in Table 1.

**TABLE 1:** Characteristics of patients with hairy cell leukaemia (HCL) and HCL‐like disorders.

	HCL	HCL‐V	SDRPL	SMZL	B‐PLL	HCL‐jv	References
Clinics							
Median age (years)	62	71	65.5	67	72	75	
Splenomegaly	+	+	+	+	+	+	
WBC ×109/L (range)		34.0 (4–346)	15.8 (3.4–36.6)		NA (26.‐1.100)	15.3 (2.81–88.8)	
Monocytopenia	+	−	−	−	−	−	
Morphology							
Abnormal lymphoid cells							
Number	Low	20%–95%	26%–91%	Low	>55%	NA	
Size	Small to medium	Medium to large	Small to medium	Small to medium	Medium	Small to medium	
Nucleolus							
Prominent	−	+	−		+	−	
Vlli							
Shape and distribution	Long, slender and circumferential	Circumferential	Unevenly distributed, polar	Short villi, polar	Absence of villi	Unevenly distributed	
Lymphoplasmocytoid cells	−	−	+	+	−	−	
Cytogenetics							
Mediane of Copy Number Aberrations (range)	3.4 (0–11)	7.9 (0–27)	NA	NA	NA	NA	23
del(17p)	Rare	42%–100%	NA	NA	NA	NA	21,22,23
Chr 5 abnormalities	7%–40%	33%	NA	NA	NA	NA	23.24
Del(7q)	21.5%	20%	NA	NA	NA	NA	23
IGHV profile							
Mutated (M)	83%	40%–73%	69.5%–79%	68%	84%	NA	22,25,39,41,54
Unmutated (UM)	10%–17%	27%–60%	21%–30.5%	32%	16%	NA	22,25,26,39,41,54
IGHV4‐34	9.4%–10%	17%–50%	15.5%	13%	2/110%	NA	27,28,31,54
IGHV1‐2*04	Rare	Rare	Rare	31%	Rare	NA	52
Genetic alterations							
BRAFV600E	93%–100%	0%	2%	2%	0%	NA	5,35
MAP2K1	Rare	50%	Rare	0%	NA	NA	32,41,50
CDKN1B (p27)	7.5%–11%	NA	NA	NA	NA	NA	34,35
CCND3	0%	13%	21%	13%	NA	NA	49
TP53	Rare	42%–100%		15%	38%	NA	27,41,54
KMT2C (MLL)	15%	0%	NA	NA	NA	NA	35
U2AF1	0%	15%	NA	NA	NA	NA	33
BCOR	NA	NA	24%	NA	25%	NA	50,54
MYD88L265P	0%–23%	NA	0%	5%–18%	25%	NA	36
KLF2	15%–23%	NA	Rare	30%	NA	NA	35
MYC	NA	NA	NA	NA	19%	NA	54
NOTCH2	Rare	Rare	Rare	10%–25%	Rare	NA	52

Abbreviations: B‐PLL, B‐prolymphocytic leukaemia; HCL, hairy cell leukaemia; HCL‐jv, Japanese form of HCL; IGHV, immunoglobulin heavy chain variable region; HCL‐V, variant form of HCL; NA, not Available; rare, less than 2 per cent; SDRPL, splenic diffuse red pulp lymphoma; SMZL, splenic marginal zone lymphoma.

### 3.1 Splenic diffuse red pulp lymphoma

Twenty‐eight years after HCL‐V, Splenic diffuse red pulp lymphoma (SDRPL) was described in 2011 in 37 patients. SDRPL appears very close to HCL‐V and SMZL.^39^ The median age of the patients was 65.5 years. The peripheral blood examination is the key element for distinguishing both entities. The infiltration consists of a large proportion of small‐ to medium‐sized cells, with round or oval nuclei and dense clumped chromatin and is homogeneous. The percentage of infiltration is high, ranging from 26% to 91% with a median of 60%. The nucleus is small or not visible in the large majority of cases and is rarely prominent. The cytoplasm is variable with well‐visible villous projections, unevenly distributed around the cell with a polar distribution. Villi are broad‐based, whereas in the others, they were rather thin. Some lymphoplasmocytic cells are often observed. Other series were later reported, describing the same features^40^, ^41^, ^42^, ^43^ with monoclonal B cells expressing CD11c (97%), less frequently CD103 (38%) and more rarely CD123 (16%) or CD25 (3%).^39^, ^44^, ^45^ ANXA1 expression was negative.

In addition, the CD200/CD180 median fluorescence intensity (MFI) ratio may be helpful to distinguish HCL from SDRPL, with a ratio of 0.5 or less suggestive of SDRPL.^15^ A scoring system based on CD11c, CD22, CD76, CD3 and CD27 was also designed to differentiate SDRPL from SMZL. One point was attributed when the CD11c relative fluorescence intensity (RFI) was higher than 25, the CD22 higher than 130, the CD76 positive, the CD27 negative and the CD38 negative. Using this scoring system, SDRPL scored 3–5 and SMZL scored 0–2.^46^

Most cases displayed a mutated *IGHV* status (86%),^47^ but the frequency ranged between 100%^40^ and 69%^41^ with overrepresentation of *IGHV4‐34* in 21% and *IGHV3‐23* in 19%.^47^

*The BRAF* V600E mutation was never detected in SDRPL. Mutations in cyclin D3 (*CCND3)* and BCL6 corepressor (*BCOR)* genes were identified in approximately a quarter of SDRPL cases. Mutations in CCND3 were identified in 24% of patients with SDRPL (6/25 pts): the expression of CCND3 was present in more than 50% of the neoplastic cells in 24/37 splenectomy specimens, while it was rarely observed in CLL, SMZL, HCL or blastic mantle cell lymphoma.^48^ Recurrent mutations or losses in BCOR were identified in 10/42 SDRPL (24%), whereas it was rarely observed in SMZL cases (2%).^49^ Neurogenic locus notch homologue protein 2 (NOTCH2) mutations (4/42 pts, 10%) were described, as well as in SMZL (8/47, 17%). Compared with SMZL, KLF2 mutations were rarely observed (2%), and TNF alpha induced protein 3 (*TNFAIP3)* and MYD88 mutations were absent in SDRPL.^49^ Other mutations in C‐X‐C motif chemokine receptor 4 (*CXCR4*) and TNF receptor‐associated factor 3 (TRAF3) were more rarely observed.^50^

### 3.2 Splenic marginal zone lymphoma

The morphology of the abnormal lymphoid cells is best appreciated in freshly made peripheral blood films. By definition, SMZL is characterized by the presence of villous lymphocytes in the peripheral blood. The percentage of villous lymphocytes is usually low and can range from 10 to 30%. Villous lymphocytes present with irregular cytoplasmic outline, short and thin villi usually concentrated at one or both poles of the cells. Their size is small or medium: the nucleus is round, showing a clumped chromatin, occasionally with prominent nucleoli. Infiltration of the bone marrow is evident in all patients: the pattern of marrow infiltration may be nodular, interstitial or diffuse. Intrasinusoidal lymphoma cells are characteristic but are not specific. Isaacson et al described in 37 patients the histopathologic features of the spleen and lymph nodes. SMZL is typically characterized by involvement of the white pulp follicles and red pulp infiltration to a varying degree with sinusoidal infiltration. Flow cytometric analysis demonstrates the expression of the B‐cell antigens CD19, CD20, CD22, CD79b and FMC7 with light chain restriction. In most cases, no reactivity was observed with CD5, CD10, CD23, CD43, CD103, CD123, myeloid and T lymphoid markers. The heavy chain is IgM with or without coexpression of IgD and less often IgG. The non‐specificity of DBA44 positivity for the distinction between HCL and SLVL was demonstrated, since abnormal HCL cells are positive and abnormal SLVL cells also positive in 78% of cases. In addition, the villous cells lack the expression of cyclin D1, Bcl‐6 and CDw75. As unusual CD5+ SMZL can be confused with leukaemic mantle cell lymphoma (MCL) associated with villous cells, splenectomy can be necessary for excluding MCL.^51^

### 3.3 B‐cell prolymphocytic leukaemia

Defined by Galton et al. in 1974 with more than 55 per cent of prolymphocytes in the peripheral blood,^4^ percentage of prolymphocytes is usually greater than 90 per cent. Peripheral blood prolymphocytes are medium‐sized cells, with moderately condensed chromatin and a single, prominent vesicular nucleolus. The nucleus is typically round or oval, and the cytoplasm is usually moderately abundant and slightly basophilic. The bone marrow is infiltrated in an interstitial or nodular pattern by similar cells.^4^ The leukaemic variant of mantle cell lymphoma can be confused morphologically with B‐PLL but is readily distinguished by the presence of t(11;14)(q13;q32) involving the cyclin D1 gene. As such, it is important to exclude this translocation, in cases of suspected B‐PLL. B‐PLL can also be confused with mixed CLL with PLL and SMZL. A complex karyotype was found in 73% of cases.^52^ The IGHV profile was mutated in 79% (15/19); 87% of patients used IGHV3 or IGHV4. The most frequent abnormality was *MYC* abnormalities (rearrangements or increased copy number) that were seen in 76 per cent of cases (26/34) and were associated with a more aggressive clinical behaviour. Deletions of 17p and *TP53* mutations were found in approximately 38 per cent (6/16). The spectrum of somatic mutations was very heterogeneous with *MYD88* (25%), *BCOR* (25%), *SF3B1* (19%) and more rarely *SETD2*, *CHD2*, *CXCR4* or *BCLAF1*. The median OS was 125.7 months for the entire cohort. The authors identified three subgroups: patients without MYC aberration (*n* = 8) with a low risk (median OS: NR), patients with MYC aberration and without del(17p) (*n* = 18) with an intermediate risk (OS: 125.7 months) and patients with both MYC and del(17p) (*n* = 7) with high‐risk (median 0S: 11.1 months).

### 3.4 Transformation/progression in others HCL‐like disorders

The patients with HCL‐like disorders may progress over the course of the disease.^3^ In SDRPL, transformation/progression of SDRPL was observed in about 11% of cases (4/37), with presence of medium to large cells.^39^ A progression rate higher occurred in 5/19 (26.3%) patients: 4/5 patients with aggressive disease had mutations in *NOTCH1* (2 cases), *TP53* (1 case) and *MAP2K1* (1 case).^41^ In the course of a review of 85 SMZL patients, four patients were subsequently classified as SDRPL with two patients presenting cutaneous involvement and all patients *TP53* mutations or anomalous p53 staining.^43^ B‐PLL developed in a patient with SDRPL^53^ and Burkitt lymphoma in a patient with B‐PLL.^54^

### 3.5 HCL Japanese

In Japan, the HCL‐JV appears much more common than HCL‐V and HCL. However, the data on HCL‐JP are very limited. In a recent meta‐analysis including only 17 patients, the median age was 75 years. The median WBC was 15.3 (2.81–88.1). The cells are small‐to‐intermediate in size and have round nuclei with inconspicuous nucleoli. Plasmacytoid appearance, characteristic of SDRPL, was absent in HCL‐JV. The proliferating cells expressed CD11c in all cases and rarely CD103 (3/12) and CD25 (1/14). ANXA1 was negative and BRAFV600E was never detected.^55^

## 4 HCL‐V/SBLPN TREATMENT

There are little specific data on the treatment of HCL‐V, but the response rate and the duration of response to PNAs were inferior to that seen in HCL.^9^ The median survival was 9 years, with 15% of patients surviving beyond 17 years.^9^ Three adverse factors emerged for a poor outcome: anaemia <10 g/dL, older age (>70 years) and the presence of *TP53* mutations. The high‐risk group of patients with one or more of these risk factors presented a 5‐year survival of 33% compared with 100% in the low risk of patients without any of these risk factors.^20^ There is no gold standard therapy neither specific guidelines for treatment of HCL‐V patients.^56^

The therapeutic approach to HCL‐V is still debated. HCL‐V is considered resistant to splenectomy, interferon, and PNAs. isolated case reports have shown the efficacy of rituximab,^57^ obinutuzumab^58^ or bendamustine plus rituximab.^59^, ^60^

In symptomatic patients, cladribine (CDA) combined with rituximab (R) was introduced by Ravandi et al.^61^ In 36 patients, including 5 HCL‐V patients, CDA was administered at 5.6 mg/m^2^ given IV over 2 h daily for 5 days and R 1 month later at 375 mg/m^2^ IV weekly for 8 weeks. With a median follow‐up of 25 months (2–68), only 1/5 patient with HCL‐V relapsed, while three HCL‐V patients died: one from relapse and 2 patients from pancreatic cancer and metastatic lung cancer, respectively. CDAR regimen was introduced later in 10 patients with CDA IV at O.15 mg/kg on days 1–5 with eight weekly doses of R IV 375 mg/m^2^ started on Day 1. Patients received a second course of R; eight weekly doses 6 months after the first course if blood minimal residual disease (MRD) were detectable. The CR rate, primary endpoint, was 90% with 8/9 patients achieving MRD‐CR at a median follow‐up of 27 months (12–48). The study was extended in 20 additional patients, including eight patients who were untreated.^62^ At 6 months after CDAR, 18/20 patients (90%) were in CR and MRD‐ according to IHC, 17/20 patients (85%) in CR and MRD‐ by FCM in the PB and 16/20 patients were in CR and MRD‐ according to IHC and FCM in the PB and BM. 11/20 patients received delayed R; nine patients becoming blood MRD+ and two patients' blood MRD positive after CDAR. The 5‐year PFS was 63.3% and the 5‐year OS was 73.9%. Ten‐year PFS and OS were 44.3% and 57.6%. Both PFS and OS were longer if blood was MRD‐ at either 1 month or 6 months. The efficacy of this treatment has also been demonstrated in a Chinese cohort of 33 HCL‐V.^63^

Ibrutinib (PCI‐32765) is a first‐in class, oral, once‐daily and irreversible BT inhibitor (BYKi)i, which covalently binds to a cysteine residue (Cys‐481) in the BTK kinase domain. Ibrutinib has been recently proven to have therapeutic activity in R/R HCL. Two R/R HCL‐V patients were treated with Ibrutinib^64^: The first one received Ibrutinib 560 mg/d in third line (first line: splenectomy, second line: R bendamustine) and achieved an unconfirmed partial response (PR) at 6 months, with a duration of response (DOR) of 16.5 months before relapsing. The relapse was unsuccessfully treated with venetoclax. The second patient received Ibrutinib 560 mg/d but it was stopped early because of gastro‐intestinal toxicities and easy bruising. The authors observed a spleen size reduction after 3 months of treatment. *Bohn* et al. also reported the rapid effectiveness of Ibrutinib 420 mg/d in a patient treated for R/R HCL‐V, with resolution of constitutional symptoms, decrease in spleen size and lymphocytosis, and increase in haemoglobin level. Despite the patient not meeting criteria for PR due to persistent thrombocytopenia, he was kept on Ibrutinib after 16 months of follow‐up, with no major toxicity.^65^ Ibrutinib was recently evaluated in a multisite phase 2 study in 37 patients with either R/R HCL (28 patients: 76%) or HCL‐V (9 patients: 24%) including 2 untreated^66^ The median number of prior treatments was 4 (range: 0–12) in HCL patients, with all of them having previously received PNAs. Patients received single‐agent ibrutinib at 420 mg daily (24 patients) or 840 mg daily (13 patients) until disease progression or unacceptable toxicity. The primary endpoint was ORR including CR and PR at 32 weeks and the best response at any time since starting ibrutinib. The ORR at 32 weeks was 24%, increasing to 36% at 48 weeks and reaching 54% at any time starting ibrutinib. One patient had a CR at 32 weeks, 8 patients a PR, 11 patients a stable disease (SD) and three patients a progressive disease (PD). Four patients were not evaluable for response. Seven patients achieved a CR as the best response at any time on study, while 13 patients had PR and 10 patients SD. Interestingly, the response rate was not statistically different between HCL and HCL‐V patients, suggesting that ibrutinib could be effective in both entities. The estimated 36‐month PFS was 73% and the estimated 36‐month OS was 85%, with no significant differences between HCL and HCL‐V. These data suggest ibrutinib could be an option in some cases of HCL and HCL‐V. Adverse events (AE) included diarrhoea (59%), myalgia (54%), nausea (51%) and bruising (43%). The frequency of cardiovascular grade 1–2 AEs was 16% for atrial fibrillation, 3% for atrial flutter, 32% for hypertension and 0%, 3% and 11%, respectively, for grade > or = 3 AEs. Unlike in CLL where the mechanism of effect of ibrutinib is well known, it is unclear in HCL. The mobilization of leukaemic cells in the peripheral blood was not observed in the majority of patients, the persistence of pERK in HCL cells was not associated with a shorter PFS and pERK cannot be used as a marker of response. Several patients with presence of pERK after 32 and 48 weeks of treatment have a durable benefit from ibrutinib. Resistance to ibrutinib is related to mutations in BTK or PLCγ2 in CLL patients: Contrary to CLL, no mutation was identified in four patients (2 HCL, 2 HCL‐V) with progressive disease, suggesting a difference in the mechanisms of resistance between HCL and CLL, despite the number of HCL patients being insufficient to establish any conclusion.

New BTKi, acalabrutinib (ACP‐196), tirabrutinib (ONO/GS‐4059), zanubrutinib (BGB‐3111) and pirtobrutinib (LOXO‐305) are being widely tested in CLL patients. Acalabrutinib, tirabrutinib and zanubrutinib are irreversible BTKi and bind irreversibly to cysteine 481 of BTK. On the contrary, pirtobrutinib is a highly selective, reversible BTKi and could be effective even in the presence of the C481S mutation of BTK. The place of these new drugs as well as that of MEK inhibitors^67^ in the treatment of HCL and HCL‐V remains to be specified.

BLC2 inhibitors (BCL2i) particularly venetoclax were used in a few atypical cases including biclonal IGHV 4–34^67^ HCL or CLL and in vitro treatment of primary hairy cells.^68^

## 5 CONCLUSION

It is essential to clearly differentiate HCL‐V3/SBLPN from HCL and others HCL‐like disorders. As SBLPN entity and the HCL‐V and B‐PLL grouping is still controversial, integrating the clinical data as well as morphologic, histologic, immunophenotypic and molecular data will allow to make in most cases a quality diagnosis and appropriate care.

## AUTHOR CONTRIBUTIONS

**Xavier Troussard:** Conceptualization (equal); formal analysis (equal); investigation (equal); methodology (equal); supervision (equal); validation (equal); visualization (equal); writing – review and editing (equal). **Elsa Maitre:** Conceptualization (equal); formal analysis (equal); methodology (equal); validation (equal); visualization (equal).

## FUNDING INFORMATION

The research received no specific grant or funding from agencies in the public, commercial or not‐for‐profit sectors.

## CONFLICT OF INTEREST STATEMENT

The authors declare they have no conflict of interest.

## Data Availability

Not applicable.
